# Integrated Transcriptomic Analysis Identifies Immune Remodeling and Prognostic Signatures in Uveal Melanoma

**DOI:** 10.1155/ijog/6511018

**Published:** 2026-02-27

**Authors:** Zhongmin Li, Youmeng Yang, Houhong Wang, Jing Wang

**Affiliations:** ^1^ Department of Ophthalmology, The Affiliated First Hospital of Fuyang Normal University, Fuyang City, Anhui Province, China; ^2^ Department of General Sugery, The Affiliated first Hospital of fuyang Normal University, Fuyang City, Anhui Province, China

**Keywords:** immune infiltration, prognostic model, risk stratification, uveal melanoma

## Abstract

**Background:**

Uveal melanoma (UVM) is the most common primary intraocular malignancy in adults and exhibits a high propensity for liver metastasis, often leading to poor prognosis. However, effective prognostic biomarkers and therapeutic strategies for metastatic UVM remain limited.

**Methods:**

We comprehensively analyzed transcriptomic data from both single‐cell and bulk RNA sequencing cohorts, integrating data from TCGA and GEO (GSE139829, GSE22138, and GSE84976). After batch effect correction and cell type annotation, differentially expressed genes (DEGs) between primary and metastatic malignant cells were identified. These were intersected with 900 prognosis‐related genes from TCGA, and 11 key prognostic genes were selected via least absolute shrinkage and selection operator (LASSO) regression to construct a risk prediction model. Model performance was evaluated across multiple cohorts. Furthermore, immune infiltration was assessed using CIBERSORT, and drug sensitivity was predicted based on chemotherapeutic IC50 values.

**Results:**

The 11‐gene risk model effectively stratified UVM patients into high‐risk and low‐risk groups with distinct survival outcomes. High‐risk patients exhibited a more immunosuppressive tumor microenvironment and were associated with altered sensitivity to multiple chemotherapeutic agents. Immune checkpoint gene expression also varied significantly between risk groups, indicating potential implications for immunotherapy response.

**Conclusion:**

This study identifies critical molecular features underlying UVM metastasis and immune remodeling, providing novel prognostic markers and potential therapeutic targets for clinical management of UVM.

## 1. Introduction

Uveal melanoma (UVM) is the most common primary intraocular malignancy in adults, originating from melanocytes within the uveal tract, which includes the iris, ciliary body, and choroid [1]. The incidence is approximately 5–7 cases per million people per year, with significantly higher rates observed in white populations, and the typical patient is a middle‐aged to elderly white individual (aged 50–70 years) [2]. Although the relationship with ultraviolet exposure remains unclear, light‐colored iris, fair skin, and sun exposure have been associated with increased risk. UVM most commonly arises in the choroid (accounting for about 85%–90%), followed by the ciliary body (5%–8%), which is associated with a poorer prognosis, and the iris (3%–5%), which is usually diagnosed earlier and has a more favorable prognosis [3]. In early stages, UVM is often asymptomatic, and as the disease progresses, symptoms such as decreased vision, visual distortion, photopsia, or visual field defects may occur [2]. Larger tumors may cause intraocular hemorrhage, secondary glaucoma, or ocular pain. On examination, fundus elevation, pigmented lesions, or masses may be observed. Current diagnostic approaches include fundus examination with slit‐lamp biomicroscopy and indirect ophthalmoscopy. Ultrasound is commonly used to assess tumor thickness, whereas optical coherence tomography (OCT) and magnetic resonance imaging (MRI) help evaluate retinal structure and local or distant invasion [4]. The primary treatments for UVM include radiotherapy and local surgical excision. Enucleation is reserved for large tumors, those causing severe vision loss, or extensive local invasion. Chemotherapy has limited efficacy, and both immunotherapy and targeted therapy remain under investigation. Approximately 50% of patients develop distant metastases within 10 years of diagnosis, most commonly to the liver, followed by the lungs and bones [5]. Once metastasis occurs, the median survival is approximately 6–12 months. Therefore, identifying effective molecular markers for early diagnosis and treatment is urgently needed.

Current treatment of UVM faces multiple challenges and difficulties, particularly in the context of effective local control but poor outcomes in the prevention and management of distant metastases. One of the most notable biological features of UVM is its strong tropism for the liver. This is attributed to the unique liver‐homing capability of UVM cells (e.g., via the CXCR4/SDF‐1 axis) and the immunosuppressive characteristics of the hepatic microenvironment [6]. Approximately 50% of patients develop metastases within 10 years of diagnosis, with about 95% presenting with liver‐only metastases. Once metastasis occurs, current therapeutic strategies fail to significantly prolong survival, with a median survival time of no more than 12 months [7]. Furthermore, UVM exhibits a low tumor mutational burden (TMB), lacks neoantigen expression, displays an immunologically “cold” tumor microenvironment, originates from an immune‐privileged site (the eye), and frequently harbors BAP1 loss‐of‐function mutations—all contributing to its extremely poor response to immune checkpoint inhibitors (ICIs) such as anti‐PD‐1 and anti‐CTLA‐4 antibodies, especially compared with cutaneous melanoma [8]. In addition, the rarity of UVM severely limits clinical trials due to the small number of eligible patients, difficulties in recruitment, low commercial returns, limited pharmaceutical investment, and a lack of suitable animal models and clinical specimens [9]. Therefore, the identification of validated serum biomarkers for early prediction and dynamic monitoring of liver metastasis is urgently needed.

The pathological staging of UVM is primarily based on the AJCC 8th edition TNM system, incorporating factors such as tumor size, ciliary body involvement, and extrascleral extension (ESE) [10]. It is classified into stages I to IV. Stage I corresponds to small, early tumors (T1a–c) without ciliary body involvement or metastasis and is associated with a favorable prognosis. Stages IIA and IIB represent medium‐sized tumors or those with ciliary body involvement or focal ESE. Stages IIIA to IIIC include larger tumors or those with significant ESE, indicating more advanced local disease. Stage IV encompasses any distant metastasis, most commonly to the liver, and carries a poor prognosis. This staging system is valuable for clinical risk assessment, therapeutic decision‐making, and follow‐up management.

Based on the above characteristics, we conducted a comprehensive investigation combining bioinformatics techniques and gene expression database, aiming to analyze the expression of genes among different clinical stages of disease progression, expecting to identify potential early predictive biomarkers and therapeutic targets for the clinical diagnosis and treatment of UVM.

## 2. Methods

### 2.1. Single‐Cell RNA Sequencing Data Analysis

In this study, we utilized the publicly available scRNA‐seq dataset GSE139829 from the GEO database, which includes primary and metastatic UVM samples. Quality control (QC) was performed to remove low‐quality cells and potential doublets. Specifically, cells with < 200 detected genes were excluded to eliminate empty droplets, while those with > 5,000 detected genes or > 25,000 unique molecular identifiers (UMIs) were removed to minimize the impact of potential doublets and multiplets. In addition, cells with a mitochondrial gene expression percentage greater than 10% were filtered out, as high mitochondrial transcript levels are widely recognized as indicators of low‐quality or apoptotic cells in scRNA‐seq analysis [11]. After QC, a total of 50,933 high‐quality single cells were retained for downstream analysis. Data preprocessing was carried out using the Seurat R package (v4.1.0), including normalization, identification of highly variable genes, centering, and scaling.

Data preprocessing was carried out using the Seurat R package (v4.1.0), including log‐normalization, identification of highly variable genes (top 2,000), centering, and scaling. To correct for batch effects among different samples, we applied the Harmony algorithm (R package v0.1.0) with the parameters theta = 2, lambda = 1, and max.iter.harmony = 20, which have been commonly used in scRNA‐seq studies to balance integration while preserving biological variability [12]. Following data integration, dimensionality reduction was performed using principal component analysis (PCA) and visualization with uniform manifold approximation and projection (UMAP). Unsupervised clustering was conducted using the Louvain algorithm at a resolution of 0.8, and the resulting cell clusters were manually annotated based on the expression of canonical marker genes.

### 2.2. Identification of Prognosis‐Related Genes

To identify key regulatory genes associated with UVM prognosis, we first performed differential gene expression analysis between primary and metastatic tumor cells at the single‐cell level using the Wilcoxon rank‐sum test, as implemented in the Seurat R package (v4.1.0). Differentially expressed genes DEGs were identified using a threshold of |log2FoldChange| > 0.25 and adjusted *p* value < 0.05. Functional enrichment analysis, including gene ontology (GO) and Kyoto Encyclopedia of Genes and Genomes (KEGG) pathway enrichment, was conducted using the ClusterProfiler R package (v4.2.2) to investigate the biological roles of DEGs.

To screen for genes significantly associated with patient prognosis, we utilized the TCGA‐UVM cohort and conducted univariate Cox regression analysis using the survival R package (v3.5‐5), identifying 900 genes with significant prognostic value (*p* < 0.05). These genes were intersected with the DEGs derived from malignant cells in the single‐cell dataset to narrow down the candidate gene set.

Subsequently, we performed LASSO regression analysis using the glmnet R package (v4.1‐2) to avoid overfitting and identify robust predictors. The optimal penalty parameter (lambda) was determined by 10‐fold cross‐validation. To ensure model stability and parsimony, we selected the lambda 1se value. This analysis resulted in the selection of 11 UVM‐specific prognostic genes, which were used to construct a polygenic risk score model. The risk score for each patient was calculated using the formula:

Risk score = *Σ* (expression level of each gene × coef).

Based on the distribution of risk scores, patients were divided into high‐risk and low‐risk groups using the median risk score as the cutoff threshold for downstream survival and clinical correlation analyses.

The prognostic performance of the model was assessed using Kaplan–Meier survival analysis (via the survminer R package, v0.4.9) and time‐dependent receiver operating characteristic (ROC) curve analysis (via the timeROC R package, v0.4), based on TCGA‐UVM data. To validate the model′s robustness and generalizability, independent validation was conducted using two external GEO datasets: GSE22138 and GSE84976.

### 2.3. Immune Infiltration and Drug Sensitivity Analysis

To investigate the immune landscape associated with different risk groups in UVM, we employed the CIBERSORT algorithm to estimate the relative proportions of 22 immune cell types from bulk RNA‐seq data in the TCGA‐UVM cohort. Gene expression profiles were normalized and formatted according to the CIBERSORT input requirements, and the LM22 signature matrix was used as a reference. The analysis was conducted using the CIBERSORT R script with 1,000 permutations to ensure robustness. Differences in immune cell infiltration between the high‐risk and low‐risk groups were evaluated using the Wilcoxon rank‐sum test.

To further explore the immunological context, we analyzed the expression patterns of a panel of immune checkpoint‐related genes (e.g., PDCD1, CTLA4, LAG3, and TIGIT) between the two groups. Statistical comparisons were performed using the Wilcoxon test to identify potential differences in immune escape or suppression mechanisms.

For drug sensitivity analysis, the pRRophetic R package (v0.5) was used to estimate the half‐maximal inhibitory concentration (IC50) of standard chemotherapeutic agents for each sample based on gene expression profiles. The algorithm applies ridge regression models trained on the Genomics of Drug Sensitivity in Cancer (GDSC) dataset to predict individual responses to drugs. We focused on commonly used antitumor drugs such as cytarabine, methotrexate, and mitomycin C. The predicted IC50 values were then compared between the high‐risk and low‐risk groups using the Wilcoxon test, aimed at assessing potential differential drug responsiveness and identifying candidate agents for risk‐group‐specific treatment.

### 2.4. Statistical Analysis

All statistical analyses were conducted using R software (Version 4.2.0). For comparisons between two groups, the Wilcoxon rank‐sum test was used for nonnormally distributed data, whereas the log‐rank test was applied for survival comparisons. Univariate Cox proportional hazards regression was performed to identify prognosis‐associated genes. LASSO regression was conducted using the glmnet package to reduce dimensionality and select key prognostic variables. Kaplan–Meier survival curves were generated using the survival and survminer packages, and time‐dependent ROC curves were plotted using the timeROC package to evaluate predictive accuracy. A two‐tailed *p* value < 0.05 was considered statistically significant unless otherwise specified.

## 3. Results

### 3.1. Single‐Cell Transcriptomic Landscape of Primary and Metastatic Uveal Melanoma

To investigate the cellular composition and transcriptomic changes in primary and metastatic UVM, we analyzed scRNA‐seq data comprising 50,933 cells from both tumor types. Batch effects were corrected using the Harmony algorithm, followed by dimensionality reduction with UMAP. The results demonstrated good integration of single‐cell data (Figure 1a), with cells from primary and metastatic UVM samples broadly distributed but partially distinguishable. Using a clustering resolution of 0.8, we identified 26 distinct cell clusters (Figure 1b). Based on the expression of classic cell type markers (Figure 1c), these clusters were ultimately annotated into seven major cell types, including malignant cells (MITF and MLANA), T/NK cells (CD3E and CD3D), monocytes/macrophages (CD14, CD68, CD163, and C1QA), B cells/plasma cells (MS4A1, CD38, and SDC1), endothelial cells (PECAM1, CD34, and CLDN5), fibroblasts (DCN, C1R, and C1S), and photoreceptor cells (RCVRN) (Figure 1d). Comparative analysis of the cellular composition between primary and metastatic tissue sources revealed a significantly increased proportion of monocytes/macrophages and T/NK cells in metastatic UVM, suggesting that the immune microenvironment in metastatic lesions had undergone remodeling (Figure 1e).

Figure 1Single‐cell transcriptomic landscape of primary and metastatic UVM. (a) UMAP visualization of 50,933 single cells from primary and metastatic UVM after batch correction using the Harmony algorithm, (b) Clustering analysis at a resolution of 0.8 identified 26 distinct cell clusters, (c) heatmap showing expression of canonical marker genes across clusters, (d) UMAP plot displays annotated major cell types, (e) bar plot showing the proportion of each cell type in primary and metastatic UVM samples, (f) volcano plot displaying differentially expressed genes between primary and metastatic malignant cells, (g) KEGG pathway enrichment of differentially expressed genes, and (h) GO enrichment analysis highlighting mitochondrial metabolic processes and ribosomal functions.

**Figure:**
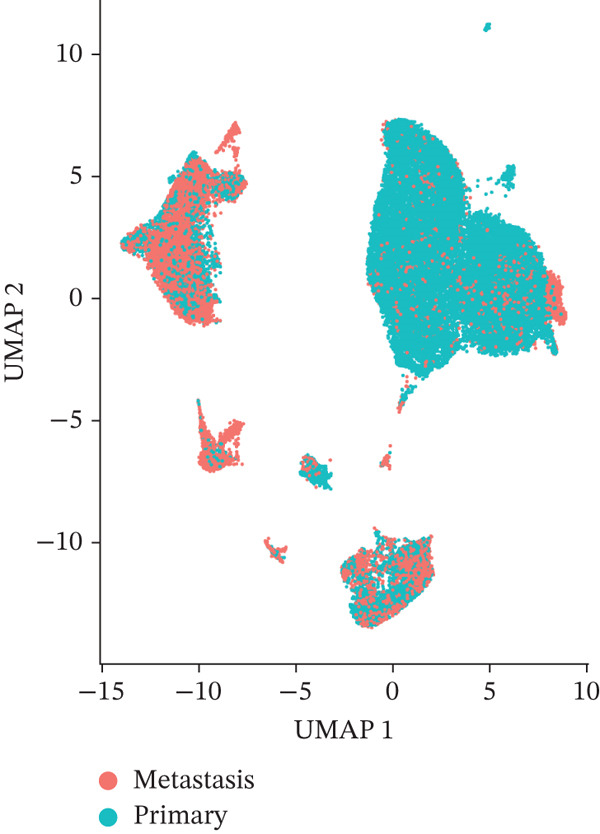
(a)

**Figure:**
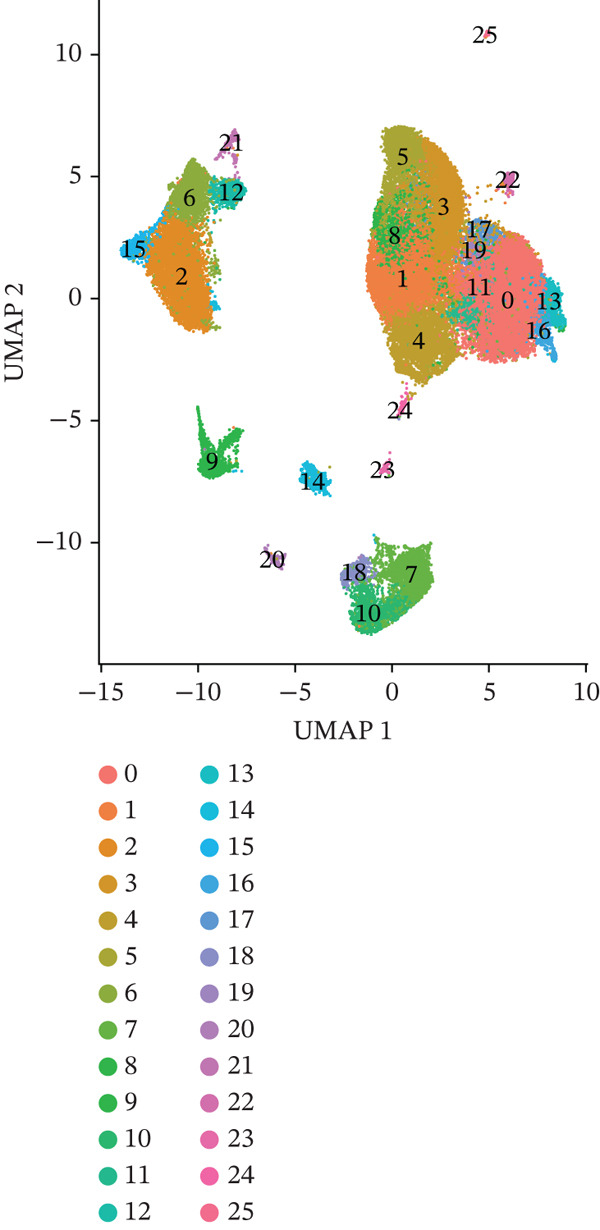
(b)

**Figure:**
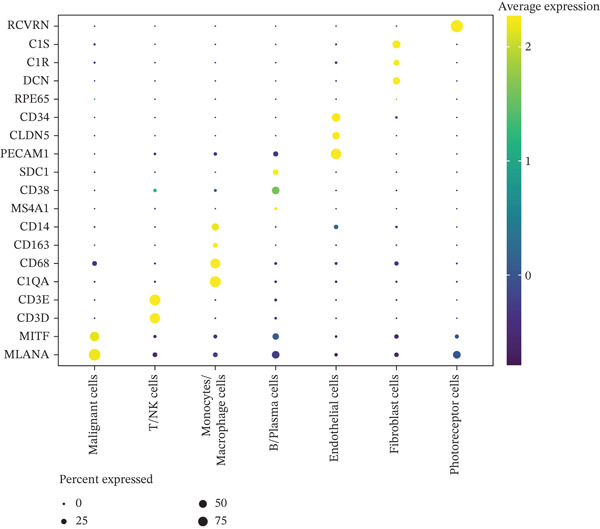
(c)

**Figure:**
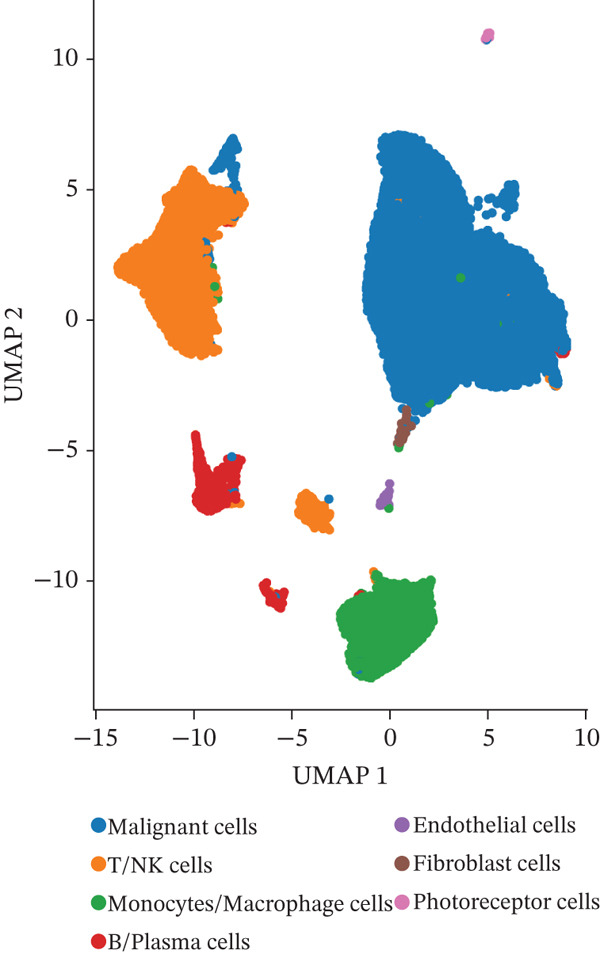
(d)

**Figure:**
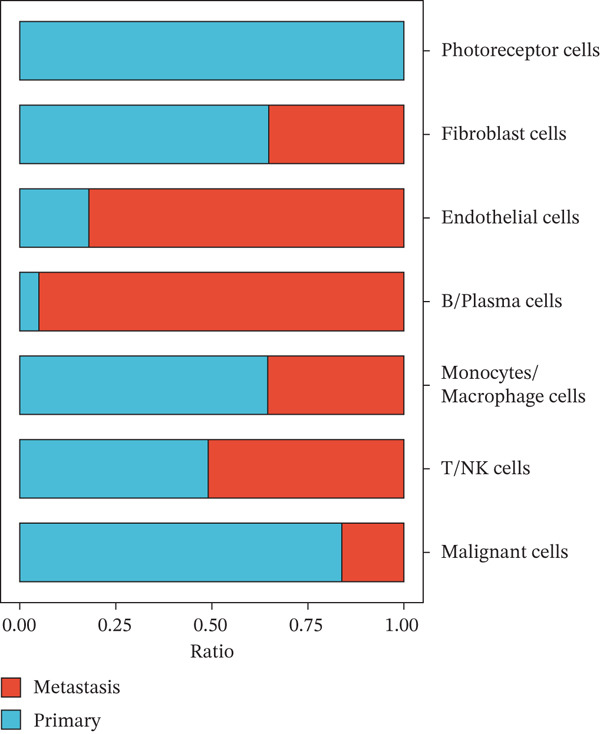
(e)

**Figure:**
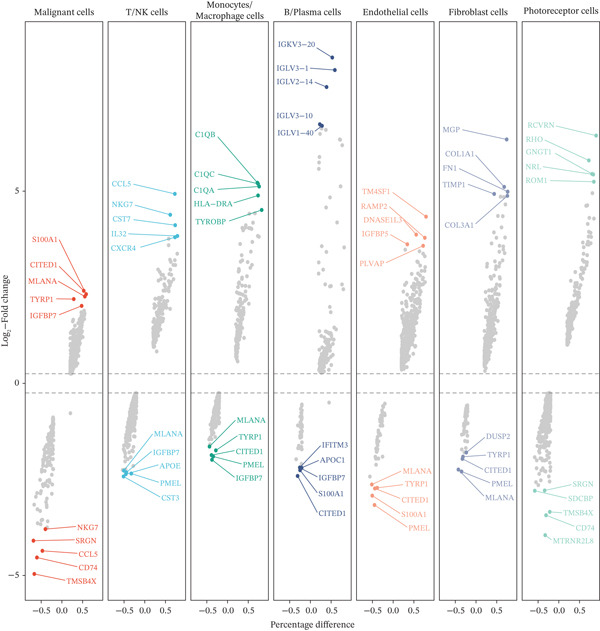
(f)

**Figure:**
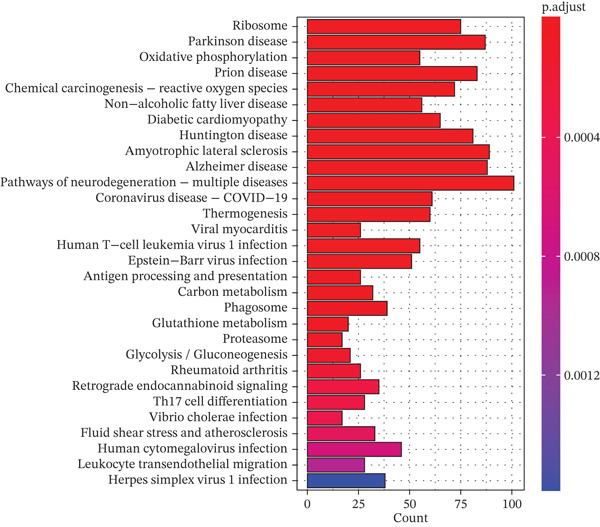
(g)

**Figure:**
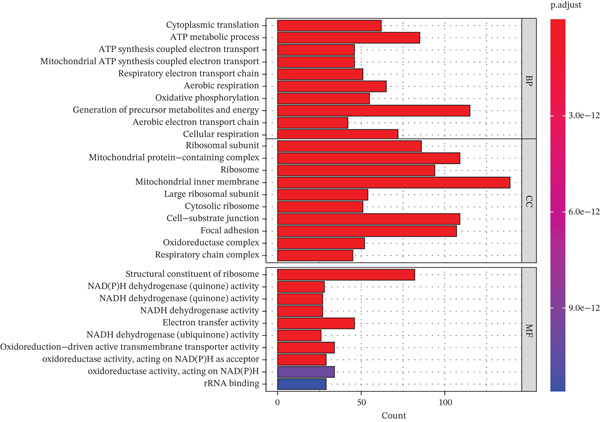
(h)

To explore changes in the intrinsic state of tumor cells, we extracted malignant cells and performed differential expression analysis. The resulting volcano plot (Figure 1f) showed multiple DEGs that were significantly upregulated or downregulated in metastatic UVM, indicating substantial transcriptional reprogramming during the metastatic process. Based on these DEGs, we conducted KEGG pathway enrichment analysis (Figure 1g), which revealed that metastatic tumor cells were enriched in pathways related to immune regulation, metabolic remodeling, and neurodegenerative diseases, including leukocyte transendothelial migration, proteasome pathway, antigen processing and presentation, and glutathione metabolism, as well as Parkinson′s disease, Alzheimer′s disease, and Huntington′s disease. These results suggest that metastatic cells may acquire an invasive phenotype through regulation of protein homeostasis, immune evasion, and oxidative stress responses. Further GO enrichment analysis (Figure 1h) showed that the DEGs were significantly involved in mitochondrial metabolism‐related processes, such as oxidative phosphorylation, ATP synthesis–coupled electron transport, and respiratory chain activity. At the cellular component level, these genes were enriched in the mitochondrial inner membrane and cytoplasmic ribosomes, whereas molecular function analysis revealed enrichment in oxidoreductase activity and NADH dehydrogenase activity.

### 3.2. Constructing a Risk Model by Integrating Bulk and Single‐Cell Transcriptome Data

To identify key regulatory factors associated with UVM prognosis, we first downloaded the TCGA‐UVM dataset and performed univariate Cox regression analysis, identifying 900 prognosis‐related genes (Table S1). These genes were then intersected with DEGs from malignant cells at the single‐cell level. The overlapping genes were subjected to LASSO regression analysis, resulting in the identification of 11 UVM‐specific prognostic genes: serpin family B member 9 (SERPINB9), poly polymerase family member 8 (PARP8), programmed cell death 4 (PDCD4), seizure related 6 homolog like 2 (SEZ6L2), KDEL endoplasmic reticulum protein retention receptor 3 (KDELR3), perilipin‐2 (PLIN2), interferon stimulated exonuclease gene 20 (ISG20), S100A13, solute carrier family 45 member 2 (SLC45A2), GABA Type A receptor associated Protein like 1 (GABARAPL1), and CTSF Cathepsin F (Table S2). As shown in Figure 2a, the LASSO coefficient trajectory plot illustrates the variation in gene coefficients under different values of the regularization parameter *λ*. Ten‐fold cross‐validation identified the optimal *λ* corresponding to the minimum partial likelihood deviance (Figure 2b). Based on the 11 selected genes, we constructed a multigene risk prediction model and calculated a risk score for each sample in the TCGA cohort. Samples were then divided into high‐risk and low‐risk groups according to the median risk score.

Figure 2Construction and validation of the UVM prognostic risk model. (a) LASSO coefficient profiles of candidate genes, (b) 10‐fold cross‐validation for optimal *λ* selection in the LASSO model, (c–d) ROC curve and Kaplan–Meier survival analysis in the TCGA‐UVM cohort, (e–f) ROC curve and survival analysis in the GSE22138 validation cohort, and (g–h) ROC curve and survival analysis in the GSE84976 validation cohort.

**Figure:**
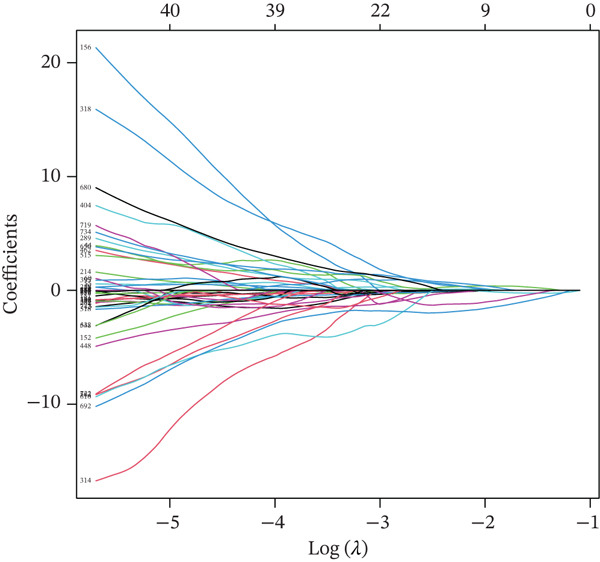
(a)

**Figure:**
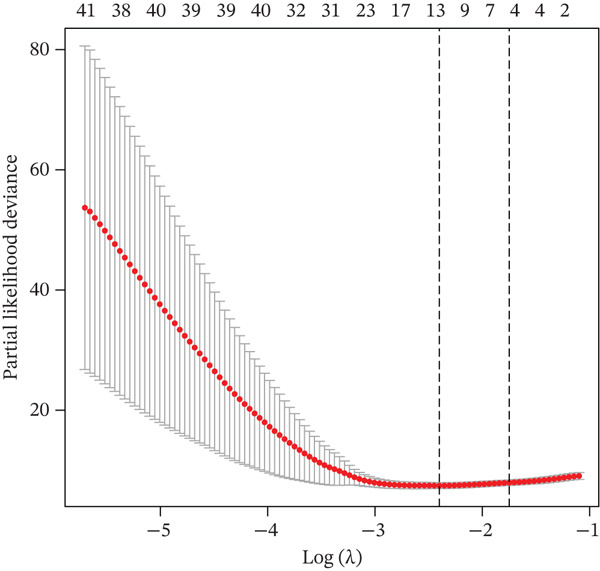
(b)

**Figure:**
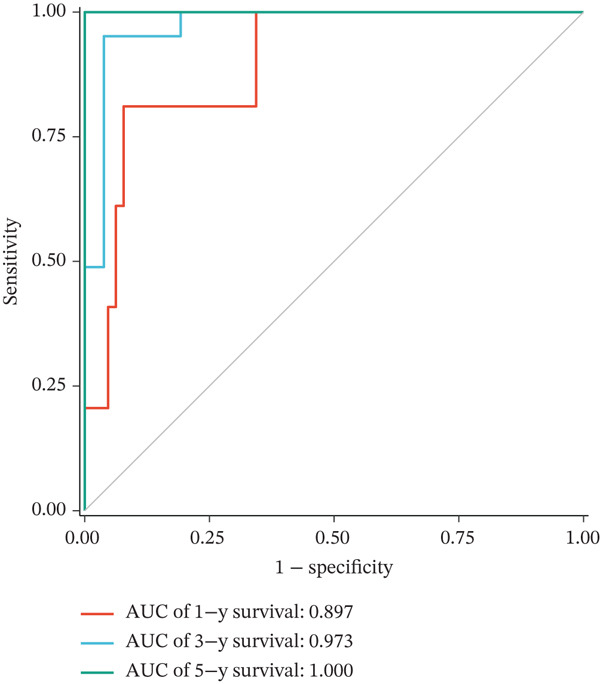
(c)

**Figure:**
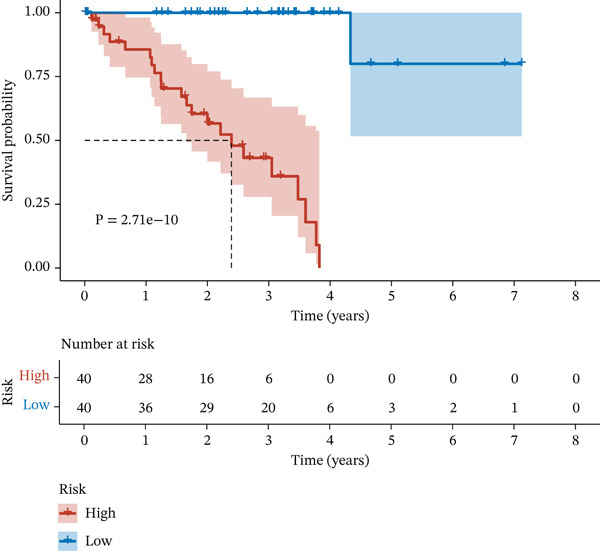
(d)

**Figure:**
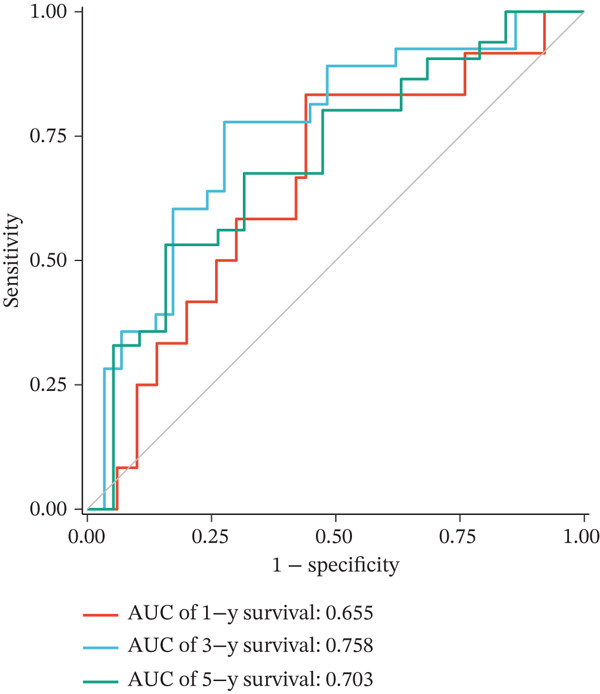
(e)

**Figure:**
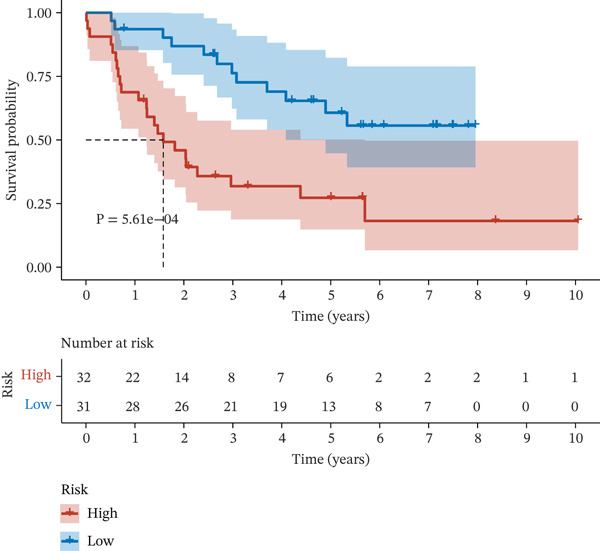
(f)

**Figure:**
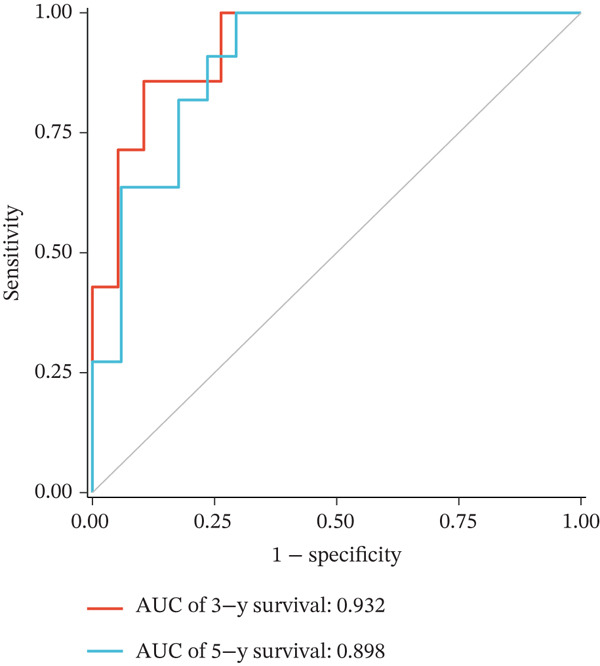
(g)

**Figure:**
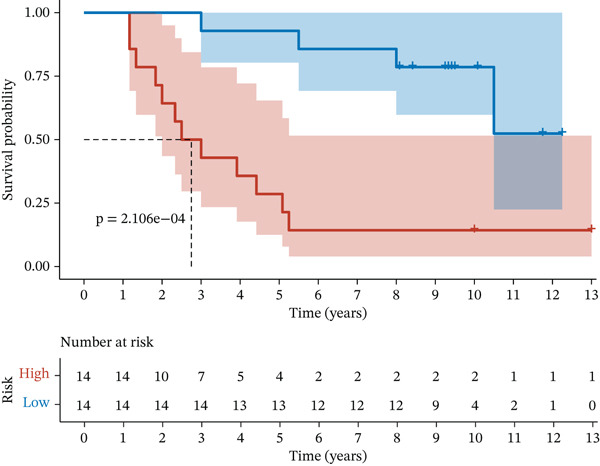
(h)

We evaluated the predictive performance of the model across multiple independent datasets. In the TCGA‐UVM training set, the model exhibited excellent predictive accuracy, with AUCs of 0.897, 0.973, and 1.000 for 1‐year, 3‐year, and 5‐year survival, respectively (Figure 2c). Kaplan–Meier survival analysis demonstrated significantly worse outcomes in the high‐risk group (*p* = 2.71e–10; Figure 2d). The model was further validated in the GSE22138 cohort, where the 1‐year, 3‐year, and 5‐year survival AUCs were 0.655, 0.758, and 0.703, respectively (Figure 2e), and high‐risk patients showed significantly poorer survival outcomes (*p* = 5.61e–04; Figure 2f). In another independent validation dataset, GSE84976, the model maintained strong predictive performance, with AUCs of 0.932 and 0.898 for 3‐year and 5‐year survival (Figure 2g), and a statistically significant difference in survival between high‐risk and low‐risk groups (*p* = 2.106e‐04; Figure 2h).

### 3.3. Prognostic Independence and Nomogram Validation of the UVM Risk Score

To evaluate whether the constructed risk model is an independent prognostic factor for overall survival (OS) in UVM patients, we performed univariate and multivariate Cox regression analyses in the TCGA‐UVM cohort. As shown in Figure 3a, univariate analysis indicated that both age (HR = 1.046, *p* = 0.019) and risk score (*H* *R* = 7.591, *p* < 0.001) were significantly associated with OS. In the multivariate model (Figure 3b), only the risk score remained an independent prognostic factor (*H* *R* = 6.983, *p* < 0.001), demonstrating the robustness of the risk model regardless of clinical covariates.

Figure 3Prognostic independence and nomogram validation of the UVM risk score. (a) Univariate Cox regression analysis of clinical variables and risk score, (b) multivariate Cox regression showing risk score as an independent prognostic factor, (c) nomogram integrating age and risk score for predicting 1‐year, 3‐year, and 5‐year overall survival, and (d) calibration curves evaluating the accuracy of nomogram‐predicted survival.

**Figure:**
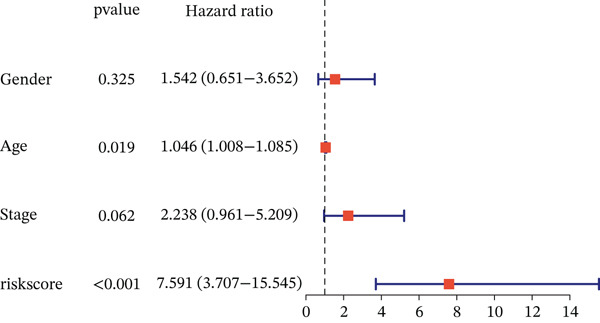
(a)

**Figure:**
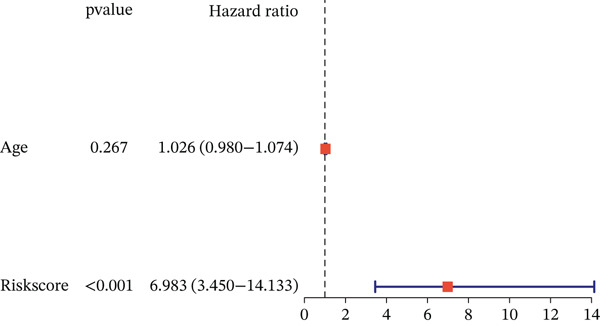
(b)

**Figure:**
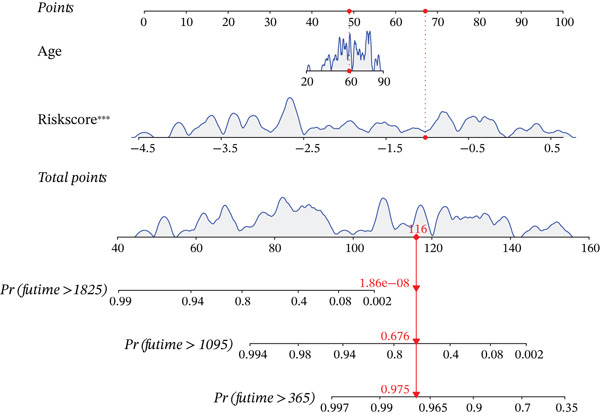
(c)

**Figure:**
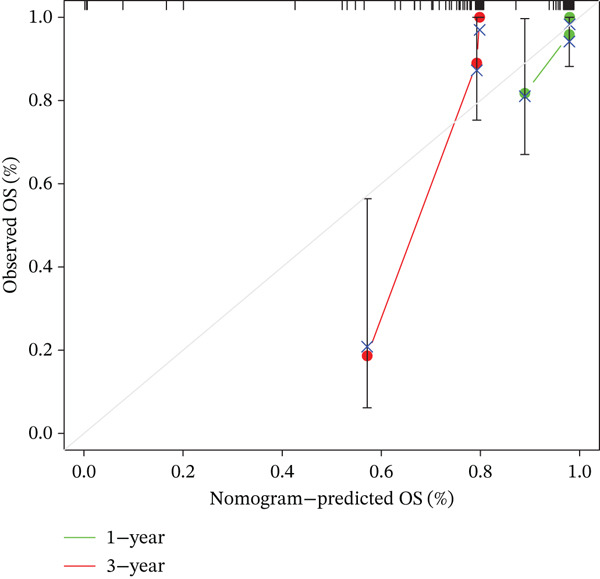
(d)

To facilitate clinical application, we constructed a nomogram integrating age and risk score to predict 1‐year, 3‐year, and 5‐year OS probabilities (Figure 3c). Each variable was assigned a point score based on its contribution to survival, and the total points corresponded to the predicted survival probability. The predictive accuracy of the nomogram was evaluated using a calibration curve (Figure 3d), which showed good agreement between the predicted and observed survival outcomes at 1 and 3 years, indicating high predictive performance and clinical utility of the risk model when combined with clinical features.

### 3.4. UVM Immune Microenvironment Characteristics and Drug Sensitivity Differences

To explore differences in the tumor immune microenvironment between high‐risk and low‐risk UVM groups, we performed CIBERSORT algorithm‐based immune infiltration analysis. As shown in Figure 4a, the infiltration levels of multiple immune cell types were significantly different between the two groups. Specifically, high‐risk patients exhibited increased enrichment of activated CD4^+^ and CD8^+^ T cells, NK cells, T helper 1 (Th1) and regulatory T (Treg) cells, MDSCs, and various antigen‐presenting cells, indicating a more immunologically active yet potentially immunosuppressive microenvironment.

Figure 4Immune characteristics and drug sensitivity analysis between high‐risk and low‐risk UVM groups. (a) Differences in immune cell infiltration levels estimated by ssGSEA (b) differential expression of immune checkpoint genes between the two risk groups, (c–e) predicted IC50 values of (c) cytarabine, (d) methotrexate, and (e) mitomycin C in high‐risk and low‐risk groups, indicating distinct drug sensitivity profiles.

**Figure:**
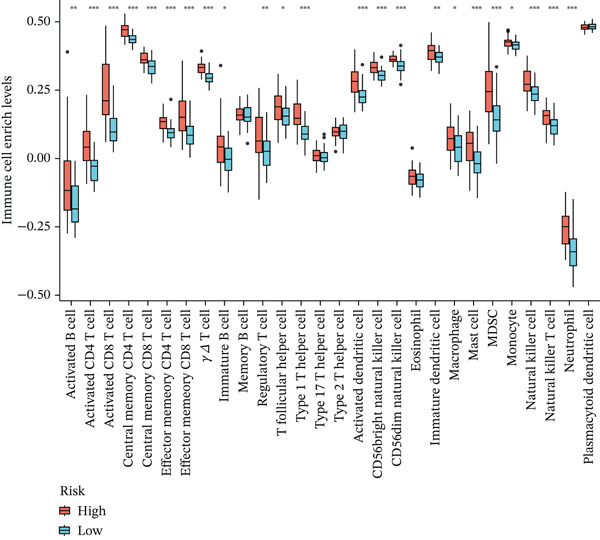
(a)

**Figure:**
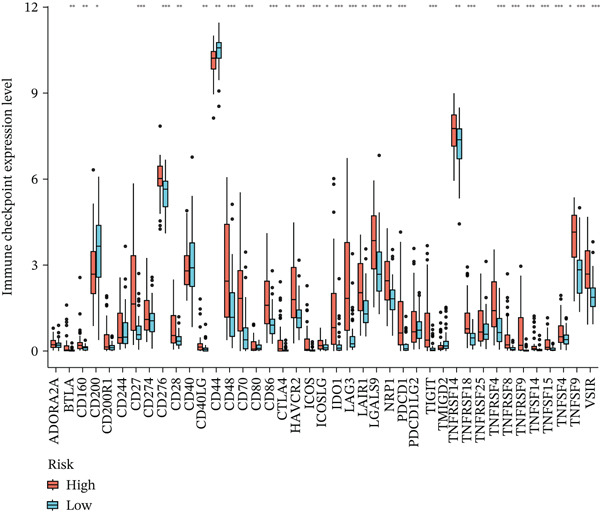
(b)

**Figure:**
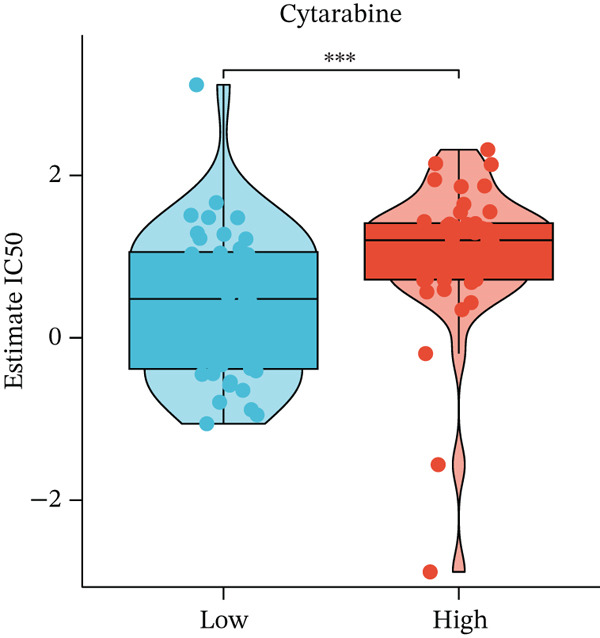
(c)

**Figure:**
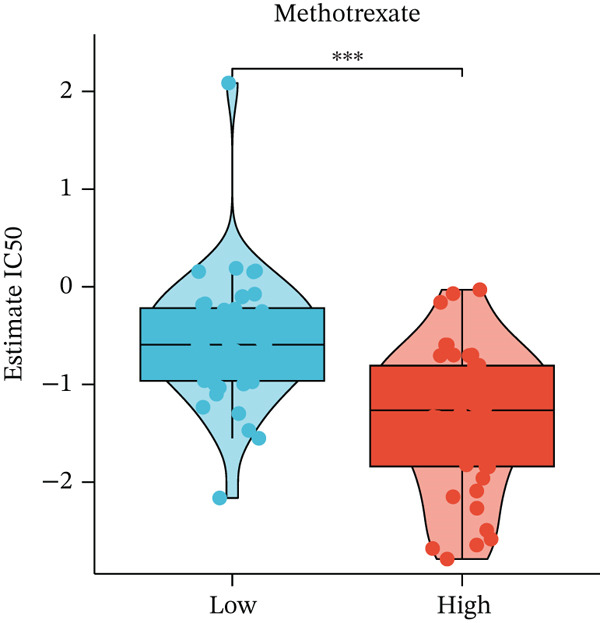
(d)

**Figure:**
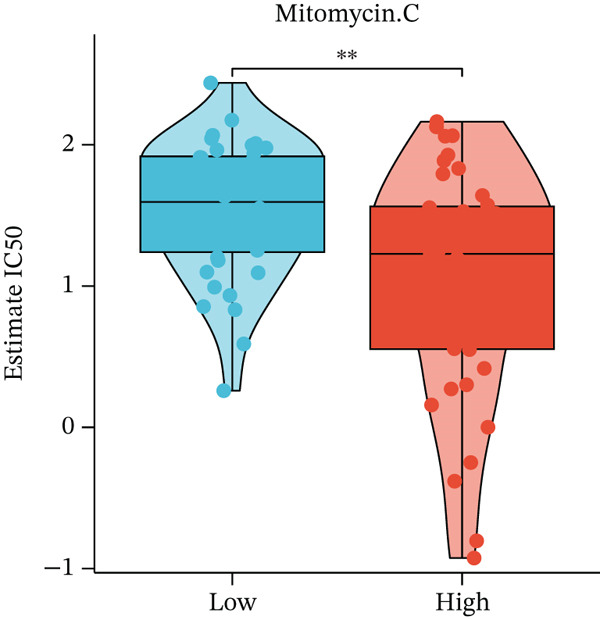
(e)

We further examined the expression of immune checkpoint genes between the two groups. As shown in Figure 4b, several immune checkpoint molecules—such as CD276 (B7‐H3), HAVCR2 (TIM‐3), LAG3, PDCD1LG2 (PD‐L2), and TIGIT—were significantly upregulated in the high‐risk group. This suggests that high‐risk patients may exhibit greater immune exhaustion and immune escape potential and may respond differently to immune checkpoint blockade (ICB) therapies.

To evaluate differences in potential drug sensitivity, we estimated the half‐maximal inhibitory concentration (IC50) of common chemotherapeutic agents. As shown in Figures 4c, 4d, and 4e, the estimated IC50 values for cytarabine and methotrexate were significantly lower in the high‐risk group (both *p* < 0.001), indicating higher sensitivity. In contrast, the IC50 of mitomycin C was significantly higher in the high‐risk group (*p* < 0.01), suggesting lower sensitivity.

### 3.5. Expression Validation of Prognostic Genes at Single‐Cell and Bulk Transcriptome Levels

To validate the expression patterns of prognostic genes across different cell types, we examined their distribution in the UVM single‐cell dataset. As shown in Figure 5a, the 11‐risk model–related genes exhibited distinct cell type–specific expression profiles. For instance, S100A13 and ISG20 were mainly expressed in endothelial and B/plasma cells, whereas SERPINB9 was highly enriched in malignant cells. PLIN2 was predominantly expressed in macrophages, and SEZ6L2 was abundant in endothelial and plasma cells. These results suggest that the prognostic genes are functionally associated with specific cell populations within the tumor microenvironment.

Figure 5Expression validation of model genes at single‐cell and bulk levels.(a) Violin plots show the expression distribution of 11 prognostic genes across UVM cell types at the single‐cell level, (b–d) Bulk RNA expression levels of (b) PLIN2, (c) S100A13, and (d) SERPINB9 in UVM samples stratified by clinical stage.

**Figure:**
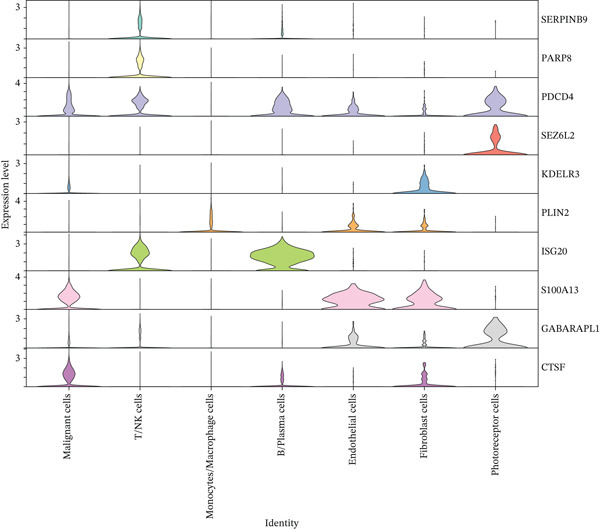
(a)

**Figure:**
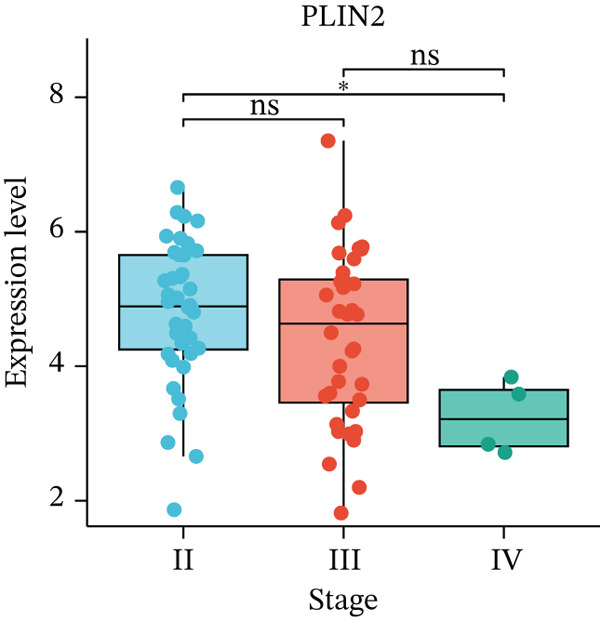
(b)

**Figure:**
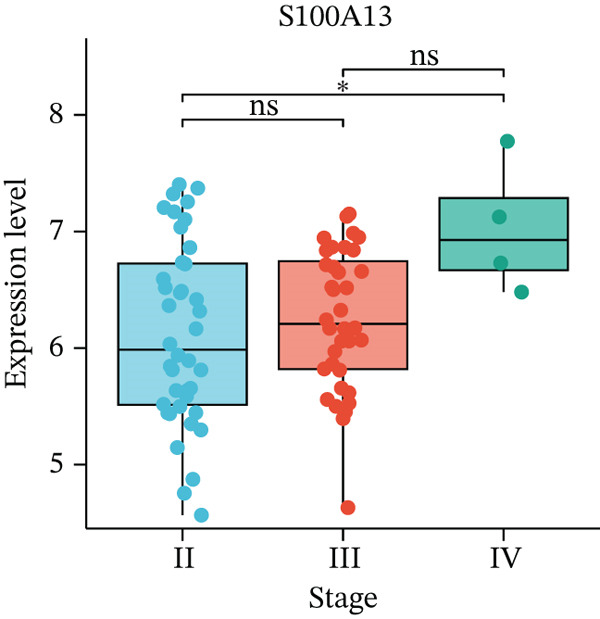
(c)

**Figure:**
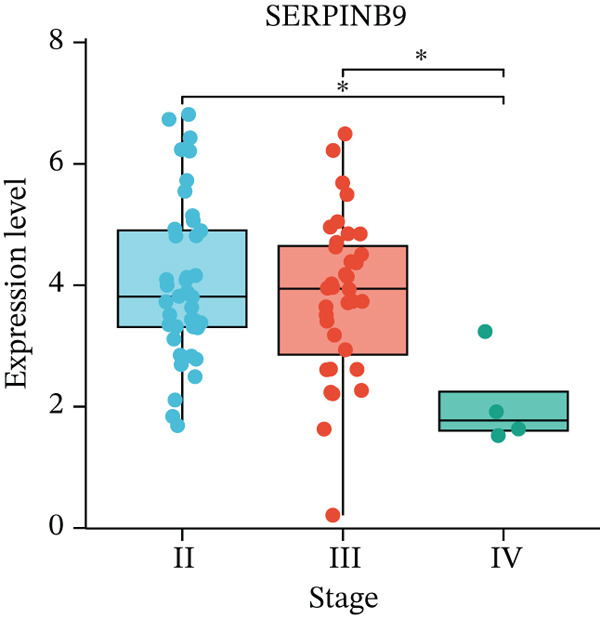
(d)

We further validated gene expression levels at the bulk transcriptomic level across UVM clinical stages (Figures 5b, 5c, and 5d). Although PLIN2 and S100A13 showed no significant differences across stages (*p* > 0.05), SERPINB9 displayed a statistically significant variation, with increased expression in Stage III tumors and decreased expression in Stage IV (*p* < 0.05), indicating its potential relevance to disease progression.

## 4. Discussion

UVM is the most common primary intraocular malignancy in adults. Although local treatment options—such as radiotherapy, local resection, and enucleation—can achieve satisfactory tumor control, significant challenges remain in clinical management [13]. The most critical issue is the high rate of distant metastasis: approximately 50% of patients develop metastases within 10 years of diagnosis, with over 95% involving the liver [14]. UVM exhibits strong hepatic tropism, and the immunosuppressive microenvironment of the liver further limits treatment efficacy. Once metastasis occurs, current therapeutic strategies fail to significantly prolong survival, with median survival typically less than 1 year. In addition, UVM has a low TMB, weak immunogenicity, and frequently harbors BAP1 loss, contributing to its poor response to ICIs, which is markedly inferior to that of cutaneous melanoma [15]. Although driver mutations such as GNAQ and GNA11 have been identified, no effective targeted therapies have been established. Research efforts are further hampered by the rarity of UVM, limited availability of clinical samples, challenges in clinical trial recruitment, and insufficient pharmaceutical investment [16]. Therefore, it is urgently necessary to develop reliable early diagnostic biomarkers and explore more effective immunotherapeutic and targeted strategies to improve long‐term outcomes for UVM patients.

In this study, we conducted a comprehensive investigation to analyze the expression of genes among different clinical stages of disease progression, and found that the expression of CTSF, GABARAPL1, ISG20, KDELR3, PARP8, PDCD4, SEZ6L2, and SLC45A2 showed no significant differences among different clinical stages of UVM. CTSF is a lysosomal cysteine protease involved in protein degradation and antigen processing, whereas ISG20 is an exonuclease that participates in antiviral responses, and higher expression of both genes has been associated with tumor invasiveness and metastasis [17, 18]. GABARAPL1 is an autophagy‐related protein that contributes to the formation and transport of autophagosomes, PARP8 belongs to the poly (ADP‐ribose) polymerase family and is involved in DNA repair and cell apoptosis, PDCD4 is a tumor suppressor gene that regulates cell proliferation, apoptosis, and transcription, which the expression levels of these three genes have been correlated with tumor growth and metastasis across various cancers [19–21]. KDELR3 is involved in protein recycling and transport between the ER and Golgi, and its high expression is linked to poor prognosis in multiple cancer types [22]. SEZ6L2 is a transmembrane protein associated with neural development and synaptic function, and its overexpression has been correlated with poor outcomes and drug resistance in several cancers [23]. SLC45A2 is a melanocyte‐associated antigen involved in melanin synthesis and transport, and is highly expressed in melanoma [24]. Mechanistically, SLC45A2 localizes to melanosomes and regulates their internal pH, which is critical for maintaining tyrosinase activity and melanin production; silencing of SLC45A2 leads to acidification of melanosomes, reduced tyrosinase activity, and decreased melanin content [25]. Studies have shown that autologous SLC45A2‐specific cytotoxic T lymphocyte (CTL) therapy demonstrates therapeutic efficacy and durability in metastatic UVM, indicating its potential as a target for immunotherapy [26]. In addition, early‐phase clinical trials of endogenous T cell (ETC) therapy targeting SLC45A2 in patients with uveal melanoma are currently underway [27].

Apart from these genes, we found the expression of PLIN2 was much higher in the Stage II subgroup compared with Stage IV patients, whereas the same differences were not shown in patients between Stage II and III as well as Stage III and IV. PLIN2, also known as adipophilin or adipocyte differentiation‐related protein (ADRP), is a key member of the lipid droplet‐associated protein family [28]. It is widely expressed in various tissues and primarily functions to regulate the formation, stabilization, and metabolism of lipid droplets [28]. PLIN2 contributes to tumor development by influencing pathways such as the cell cycle, ferroptosis, endoplasmic reticulum stress, and reactive oxygen species generation [29]. Studies have shown that the expression level of PLIN2 is closely correlated with the survival rate of patients with UVM [30]. Furthermore, changes in PLIN2 expression are linked to immunosuppression and metabolic reprogramming within the tumor microenvironment [30]. Meanwhile, we found the expression of S100A13 showed the same trend in patients with UVM. S100A13 is a member of the S100 protein family and is widely distributed in both the cytoplasm and nucleus. It is involved in regulating various cellular processes, including cell cycle progression, differentiation, inflammatory responses, and cell migration [31]. In several types of cancer, such as non–small cell lung cancer, high expression of S100A13 has been associated with enhanced metastatic potential and poor patient prognosis [32, 33]. Notably, recent studies on other S100 family members have further revealed the family′s pivotal role in inflammation‐related tissue injury and immune regulation. For example, studies have reported that S100A8/A9‐overexpressing macrophages mediate renal tubular epithelial cell injury in acute kidney injury following acute Type A aortic dissection surgery, suggesting the significant role of S100 family proteins in shaping the inflammatory microenvironment and regulating disease progression [34]. Although S100A8/A9 and S100A13 exhibit functional differences, they share structural similarities and participate in overlapping inflammatory signaling pathways. This suggests that abnormal expression of S100 family members may contribute to pathological remodeling through immune‐related mechanisms. However, previous studies have not investigated the relationship between S100A13 and UVM. This study first reveals the expression profile of S100A13 in UVM and its potential significance, expanding the functional spectrum of S100 family proteins in disease.

Additionally, we also found the expression of SERPINB9 showed significant differences among Stage II, III, and IV subgroups. SERPINB9 is a serine protease inhibitor that primarily functions to prevent apoptosis by inhibiting granzyme B released by immune cells [35]. Studies have shown that high expression of SERPINB9 is associated with poor prognosis in patients with UVM [36]. It impairs CTL and natural killer (NK) cell‐mediated tumor cell apoptosis, thereby promoting immune evasion [37, 38]. Furthermore, research has demonstrated that knockout of SERPINB9 enhances tumor cell susceptibility to T cell‐mediated cytotoxicity, suggesting its potential as a therapeutic target [37]. Notably, several studies have indicated that SERPINB9 contributes to tumor immune evasion through multiple mechanisms. In a pancancer analysis, Ibáñez‐Molero and colleagues identified SERPINB9 as the most DEG in tumor cells resistant to ICB therapy. Its expression was further elevated in melanoma samples following ICB treatment, and rare SERPINB9‐positive cells were also detected in non–small cell lung cancer [39]. In functional assays, Jiang′s group demonstrated that genetic deletion or pharmacological inhibition of SERPINB9 markedly increased tumor cell susceptibility to T cell‐ and CAR‐T cell–mediated cytotoxicity [40]. Complementing these findings, a clinical cohort study by Soriano et al. reported that tumor cells frequently upregulate PI‐9/SERPINB9 as a defense against granzyme B–dependent cytotoxicity [41]. Together, these observations highlight a consistent role of SERPINB9 in facilitating immune escape and underscore its potential as a therapeutic target in UVM.

There also remain several limitations in our study. Firstly, as this work is based on publicly available transcriptomic datasets, the innate technical constraints of bioinformatics analysis may introduce potential bias. Secondly, our immune infiltration and drug sensitivity analyses were derived from computational predictions based on the 11‐gene risk model. These stratified trends cannot be directly replicated by in vitro assays, and clinical validation would require additional tumor tissue collection and multigene testing, which are not currently feasible in routine UVM practice. Thirdly, deeper mechanistic studies using cellular or animal models are warranted to further verify the biological functions of the model genes and to strengthen the translational significance of our findings.

In conclusion, our comprehensive investigation combined bioinformatics techniques and gene expression databases, aimed at analyzing the expression of genes among different clinical stages of disease progression, and found significant expression differences of PLIN2, S100A13, and SERPINB9, which could be potential early predictive biomarkers and therapeutic targets for the clinical diagnosis and treatment of UVM.

## Author Contributions

Zhongmin Li and Youmeng Yang contributed to the original draft writing, validation, formal analysis, visualization, software development, methodology design, conceptualization, and data curation. Houhong Wang and Jing Wang were responsible for methodology support, manuscript review and editing, funding acquisition, resource provision, supervision, and project administration.

## Funding

No funding was received for this manuscript.

## Disclosure

All authors contributed to data analysis, drafting, or revising of the article, agree on the journal to which the article is being submitted, provided final approval of the version to be published, and agree to be accountable for all aspects of the work.

## Ethics Statement

The authors have nothing to report.

## Consent

Prior to the commencement of data collection, the corresponding author obtained written consent from all participants concerning participation and subsequent publication of the study results.

## Conflicts of Interest

The authors declare no conflicts of interest.

## Supporting information


**Supporting Information** Additional supporting information can be found online in the Supporting Information section. Table S1 900 prognosis‐related genes identified by uniCOX in TCGA datasets Table S2: 11 UVM‐specific prognostic genes selected by LASSO algorithm.

## Data Availability

The gene expression datasets analyzed in this study are publicly available in the NCBI Gene Expression Omnibus (GEO). Data were obtained from GEO under accession numbers. GSE139829 (https://www.ncbi.nlm.nih.gov/geo/query/acc.cgi?acc=GSE139829), GSE22138 (https://www.ncbi.nlm.nih.gov/geo/query/acc.cgi?acc=GSE22138), GSE84976 (https://www.ncbi.nlm.nih.gov/geo/query/acc.cgi?acc=GSE84976).

## References

[1] Chattopadhyay C. , Kim D. W. , Gombos D. S. , Oba J. , Qin Y. , Williams M. D. , Esmaeli B. , Grimm E. A. , Wargo J. A. , Woodman S. E. , and Patel S. P. , Uveal Melanoma: From Diagnosis to Treatment and the Science in Between, Cancer. (2016) 122, no. 15, 2299–2312, 10.1002/cncr.29727, 2-s2.0-84961226271, 26991400. 26991400 PMC5567680

[2] Kaliki S. and Shields C. L. , Uveal Melanoma: Relatively Rare but Deadly Cancer, Eye (London, England). (2017) 31, no. 2, 241–257, 10.1038/eye.2016.275, 2-s2.0-85021455051, 27911450. 27911450 PMC5306463

[3] Jager M. J. , Shields C. L. , Cebulla C. M. , Abdel-Rahman M. H. , Grossniklaus H. E. , Stern M.-H. , Carvajal R. D. , Belfort R. N. , Jia R. , Shields J. A. , and Damato B. E. , Uveal Melanoma, Nature Reviews Disease Primers. (2020) 6, no. 1, 10.1038/s41572-020-0158-0. 32273508

[4] Kheir W. J. , Kim J. S. , and Materin M. A. , Multiple Uveal Melanoma, Ocular Oncology and Pathology. (2020) 6, no. 5, 368–375, 10.1159/000508393, 33123531. 33123531 PMC7574613

[5] Weis E. , Surgeoner B. , Salopek T. G. , Cheng T. , Hyrcza M. , Kostaras X. , Larocque M. , McKinnon G. , McWhae J. , Menon G. , Monzon J. , Murtha A. D. , Walker J. , and Temple-Oberle C. , Management of Uveal Melanoma: Updated Cancer Care Alberta Clinical Practice Guideline, Current Oncology (Toronto, Ont.). (2023) 31, no. 1, 24–41, 10.3390/curroncol31010002, 38275828. 38275828 PMC10814960

[6] Robertson A. G. , Shih J. , Yau C. , Gibb E. A. , Oba J. , Mungall K. L. , Hess J. M. , Uzunangelov V. , Walter V. , Danilova L. , Lichtenberg T. M. , Kucherlapati M. , Kimes P. K. , Tang M. , Penson A. , Babur O. , Akbani R. , Bristow C. A. , Hoadley K. A. , Iype L. , Chang M. T. , Cherniack A. D. , Benz C. , Mills G. B. , Verhaak R. G. W. , Griewank K. G. , Felau I. , Zenklusen J. C. , Gershenwald J. E. , Schoenfield L. , Lazar A. J. , Abdel-Rahman M. H. , Roman-Roman S. , Stern M.-H. , Cebulla C. M. , Williams M. D. , Jager M. J. , Coupland S. E. , Esmaeli B. , Kandoth C. , and Woodman S. E. , Integrative Analysis Identifies Four Molecular and Clinical Subsets in Uveal Melanoma, Cancer Cell. (2018) 33, no. 1, 10.1016/j.ccell.2017.12.013, 2-s2.0-85045090027. 29316429

[7] Wespiser M. , Neidhardt E. , and Negrier S. , Uveal Melanoma: In the Era of New Treatments, Cancer Treatment Reviews. (2023) 119, 102599, 10.1016/j.ctrv.2023.102599, 37473516. 37473516

[8] Singh N. , Singh R. , Bowen R. C. , Abdel-Rahman M. H. , and Singh A. D. , Uveal Melanoma in BAP1 Tumor Predisposition Syndrome: Estimation of Risk, American Journal of Ophthalmology. (2021) 224, 172–177, 10.1016/j.ajo.2020.12.005, 33316260. 33316260 PMC8059106

[9] Bakhoum M. F. , Curtis E. J. , Goldbaum M. H. , and Mischel P. S. , BAP1 Methylation: A Prognostic Marker of Uveal Melanoma Metastasis, Npj Precision Oncology. (2021) 5, no. 1, 10.1038/s41698-021-00226-8, 34593944. PMC8484429 34593944

[10] Carvajal R. D. , Sacco J. J. , Jager M. J. , Eschelman D. J. , Olofsson Bagge R. , Harbour J. W. , Chieng N. D. , Patel S. P. , Joshua A. M. , and Piperno-Neumann S. , Advances in the Clinical Management of Uveal Melanoma, Clinical Oncology. (2023) 20, no. 2, 99–115, 10.1038/s41571-022-00714-1. 36600005

[11] Luecken M. D. and Theis F. J. , Current Best Practices in Single-Cell RNA-Seq Analysis: A Tutorial, Molecular Systems Biology. (2019) 15, no. 6, e8746, 10.15252/msb.20188746, 2-s2.0-85067863532. 31217225 PMC6582955

[12] Korsunsky I. , Millard N. , Fan J. , Slowikowski K. , Zhang F. , Wei K. , Baglaenko Y. , Brenner M. , Loh P. R. , and Raychaudhuri S. , Fast, Sensitive and Accurate Integration of Single-Cell Data With Harmony, Nature Methods. (2019) 16, no. 12, 1289–1296, 10.1038/s41592-019-0619-0, 31740819. 31740819 PMC6884693

[13] Chen L. N. and Carvajal R. D. , Tebentafusp for the Treatment of HLA-A∗02: 01-Positive Adult Patients With Unresectable or Metastatic Uveal Melanoma, Expert Review of Anticancer Therapy. (2022) 22, no. 10, 1017–1027, 10.1080/14737140.2022.2124971, 36102132. 36102132 PMC10184536

[14] Bustamante P. , Piquet L. , Landreville S. , and Burnier J. V. , Uveal Melanoma Pathobiology: Metastasis to the Liver, Seminars in Cancer Biology. (2021) 71, 65–85, 10.1016/j.semcancer.2020.05.003, 32450140. 32450140

[15] Masoomian B. , Shields J. A. , and Shields C. L. , Overview of BAP1 Cancer Predisposition Syndrome and the Relationship to Uveal Melanoma, Journal of Current Ophthalmology. (2018) 30, no. 2, 102–109, 10.1016/j.joco.2018.02.005, 2-s2.0-85044326335, 29988936. 29988936 PMC6034168

[16] Bai H. , Bosch J. J. , and Heindl L. M. , Current Management of Uveal Melanoma: A Review, Clinical & Experimental Ophthalmology. (2023) 51, no. 5, 484–494, 10.1111/ceo.14214, 37076276. 37076276

[17] Chai G. , Li S. , Yang Y. , Zhou G. , and Wang Y. , CTSF An Intrusion Detection Framework for Industrial Internet Based on Enhanced Feature Extraction and Decision Optimization Approach, Sensors. (2023) 23, no. 21, 10.3390/s23218793, 37960495. PMC10647644 37960495

[18] Jia M. , Li L. , Chen R. , Du J. , Qiao Z. , Zhou D. , Liu M. , Wang X. , Wu J. , Xie Y. , Sun Y. , Zhang Y. , Wang Z. , Zhang T. , Hu H. , Sun J. , Tang W. , and Yi F. , Targeting RNA Oxidation by ISG20-Mediated Degradation Is a Potential Therapeutic Strategy for Acute Kidney Injury, Molecular Therapy: The Journal of the American Society of Gene Therapy. (2023) 31, no. 10, 3034–3051, 10.1016/j.ymthe.2023.07.008, 37452495. 37452495 PMC10556188

[19] Jacquet M. , Hervouet E. , Baudu T. , Herfs M. , Parratte C. , Feugeas J.-P. , Perez V. , Reynders C. , Ancion M. , Vigneron M. , Baguet A. , Guittaut M. , Fraichard A. , and Despouy G. , GABARAPL1 Inhibits EMT Signaling Through SMAD-Tageted Negative Feedback, Biology. (2021) 10, no. 10, 10.3390/biology10100956. PMC8533302 34681055

[20] Alfahed A. , Ebili H. O. , Almoammar N. E. , Alasiri G. , AlKhamees O. A. , Aldali J. A. , Al Othaim A. , Hakami Z. H. , Abdulwahed A. M. , and Waggiallah H. A. , Prognostic Values of Gene Copy Number Alterations in Prostate Cancer, Genes. (2023) 14, no. 5, 10.3390/genes14050956, 37239316. PMC10217731 37239316

[21] Cai Q. , Yang H.-S. , Li Y.-C. , and Zhu J. , Dissecting the Roles of PDCD4 in Breast Cancer, Frontiers in Oncology. (2022) 12, 855807, 10.3389/fonc.2022.855807, 35795053. 35795053 PMC9251513

[22] Yuan J. , Zhuang Y.-Y. , Liu X. , Zhang Y. , Li K. , Chen Z. J. , Li D. , Chen H. , Liang J. , Yao Y. , Yu X. , Zhuo R. , Zhao F. , Zhou X. , Yu X. , Qu J. , and Su J. , Exome-Wide Association Study Identifies KDELR3 Mutations in Extreme Myopia, Nature Communications. (2024) 15, no. 1, 10.1038/s41467-024-50580-x, 39112444. PMC11306401 39112444

[23] Abe M. , Yaguchi H. , Kudo A. , Nagai A. , Shirai S. , Takahashi-Iwata I. , Matsushima M. , Nakamura N. , Isahaya K. , Yamano Y. , Ashida S. , Kasai T. , Tanaka K. , Watanabe M. , Kondo T. , Takahashi H. , Hatakeyama S. , Takekoshi A. , Kimura A. , Shimohata T. , and Yabe I. , Sez6l2 Autoimmunity in a Large Cohort Study, Journal of Neurology, Neurosurgery, and Psychiatry. (2023) 94, no. 8, 667–668, 10.1136/jnnp-2022-330194, 37263766. 37263766

[24] Liu Y. , Chi W. , Tao L. , Wang G. , Deepak R. N. V. K. , Sheng L. , Chen T. , Feng Y. , Cao X. , Cheng L. , Zhao X. , Liu X. , Deng H. , Fan H. , Jiang P. , and Chen L. , Ablation of Proton/Glucose Exporter SLC45A2 Enhances Melanosomal Glycolysis to Inhibit Melanin Biosynthesis and Promote Melanoma Metastasis, Journal of Investigative Dermatology. (2022) 142, no. 10, 2744–2755.e9, 10.1016/j.jid.2022.04.008. 35469906

[25] Bin B. H. , Bhin J. , Yang S. H. , Shin M. , Nam Y. J. , Choi D. H. , Shin D. W. , Lee A. Y. , Hwang D. , Cho E. G. , and Lee T. R. , Membrane-Associated Transporter Protein (MATP) Regulates Melanosomal pH and Influences Tyrosinase Activity, PLoS One. (2015) 10, no. 6, e0129273, 10.1371/journal.pone.0129273, 2-s2.0-84936803092. 26057890 PMC4461305

[26] Park J. , Talukder A. H. , Lim S. A. , Kim K. , Pan K. , Melendez B. , Bradley S. D. , Jackson K. R. , Khalili J. S. , Wang J. , Creasy C. , Pan B.-F. , Woodman S. E. , Bernatchez C. , Hawke D. , Hwu P. , Lee K.-M. , Roszik J. , Lizée G. , and Yee C. , SLC45A2: A Melanoma Antigen With High Tumor Selectivity and Reduced Potential for Autoimmune Toxicity, Cancer Immunology Research. (2017) 5, no. 8, 618–629, 10.1158/2326-6066.CIR-17-0051, 2-s2.0-85026747911, 28630054. 28630054 PMC6087543

[27] Zhou Y. , Cao Y. , Li Z. , Lei X. , Gao Q. , Yao W. , Guan J. , Lu G. , Deng H. , Zhang L. , Deng X. , Chen Z. , and Xing Y. , Single-Cell Profiling Deciphering Cholesterol Metabolism Dysregulation in Metastatic Uveal Melanoma and Implicating SLC45A2 in its Prognosis, Frontiers in Immunology. (2025) 16, 1660268, 10.3389/fimmu.2025.1660268, 41103423. 41103423 PMC12521093

[28] Roberts M. A. , Deol K. K. , Mathiowetz A. J. , Lange M. , Leto D. E. , Stevenson J. , Hashemi S. H. , Morgens D. W. , Easter E. , Heydari K. , Nalls M. A. , Bassik M. C. , Kampmann M. , Kopito R. R. , Faghri F. , and Olzmann J. A. , Parallel CRISPR-Cas9 Screens Identify Mechanisms of PLIN2 and Lipid Droplet Regulation, Developmental Cell. (2023) 58, no. 18, 1782–1800.e10, 10.1016/j.devcel.2023.07.001, 37494933. 37494933 PMC10530302

[29] Wu Y. , Chen K. , Li L. , Hao Z. , Wang T. , Liu Y. , Xing G. , Liu Z. , Li H. , Yuan H. , Lu J. , Zhang C. , Zhang J. , Zhao D. , Wang J. , Nie J. , Ye D. , Pan G. , Chan W.-Y. , and Liu X. , Plin2-Mediated Lipid Droplet Mobilization Accelerates Exit From Pluripotency by Lipidomic Remodeling and Histone Acetylation, Cell Death and Differentiation. (2022) 29, no. 11, 2316–2331, 10.1038/s41418-022-01018-8, 35614132. 35614132 PMC9613632

[30] Matareed M. , Maranou E. , Koskela S. A. , Mehmood A. , Kalirai H. , Coupland S. E. , and Figueiredo C. R. , Novel Prognostication Biomarker Adipophilin Reveals a Metabolic Shift in Uveal Melanoma and New Therapeutic Opportunities, Journal of Pathology. (2023) 260, no. 2, 203–221, 10.1002/path.6076, 36825655. 36825655

[31] Su Y. , Xu C. , Sun Z. , Liang Y. , Li G. , Tong T. , and Chen J. , S100A13 Promotes Senescence-Associated Secretory Phenotype and Cellular Senescence Via Modulation of Non-Classical Secretion of IL-1*α* , Aging. (2019) 11, no. 2, 549–572, 10.18632/aging.101760, 2-s2.0-85060942734, 30670674. 30670674 PMC6366962

[32] Al-Mutairy E. A. , Al Qattan S. , Khalid M. , Al-Enazi A. A. , Al-Saif M. M. , Imtiaz F. , Ramzan K. , Raveendran V. , Alaiya A. , Meyer B. F. , Atamas S. P. , Collison K. S. , Khabar K. S. , Hasday J. D. , and Al-Mohanna F. , Wild-Type S100A3 and S100A13 Restore Calcium Homeostasis and Mitigate Mitochondrial Dysregulation in Pulmonary Fibrosis Patient-Derived Cells, Frontiers in Cell and Developmental Biology. (2023) 11, 1282868, 10.3389/fcell.2023.1282868, 38099297. 38099297 PMC10720433

[33] Miao S. , Qiu T. , Zhao Y. , Wang H. , Sun X. , Wang Y. , Xuan Y. , Qin Y. , and Jiao W. , Overexpression of S100A13 Protein Is Associated With Tumor Angiogenesis and Poor Survival in Patients With Early-Stage Non-Small Cell Lung Cancer, Thoracic Cancer. (2018) 9, no. 9, 1136–1144, 10.1111/1759-7714.12797, 2-s2.0-85052579253, 30047626. 30047626 PMC6119616

[34] Cai X. , Li X. , Shi J. , Tang L. , Yang J. , Yu R. , Wang Z. , and Wang D. , S100A8/A9 High-Expression Macrophages Mediate Renal Tubular Epithelial Cell Damage in Acute Kidney Injury Following Acute Type a Aortic Dissection Surgery, Frontiers in Molecular Biosciences. (2025) 12, 1530741, 10.3389/fmolb.2025.1530741, 40270593. 40270593 PMC12015165

[35] Bird C. H. , Sutton V. R. , Sun J. , Hirst C. E. , Novak A. , Kumar S. , Trapani J. A. , and Bird P. I. , Selective regulation of apoptosis: the cytotoxic lymphocyte serpin proteinase inhibitor 9 (PI-9) inhibits granzyme B, Journal of Biological Chemistry. (1998) 273, 294–299. 10.1128/mcb.18.11.6387 PMC109224 9774654

[36] Huang H. , Mu Y. , and Li S. , The Biological Function of Serpinb9 and Serpinb9-Based Therapy, Frontiers in Immunology. (2024) 15, 1422113, 10.3389/fimmu.2024.1422113, 38966643. 38966643 PMC11222584

[37] Jiang P. , Gu S. , Pan D. , Fu J. , Sahu A. , Hu X. , Li Z. , Traugh N. , Bu X. , Li B. , Liu J. , Freeman G. J. , Brown M. A. , Wucherpfennig K. W. , and Liu X. S. , Signatures of T Cell Dysfunction and Exclusion Predict Cancer Immunotherapy Response, Nature Medicine. (2018) 24, no. 10, 1550–1558, 10.1038/s41591-018-0136-1, 2-s2.0-85052583273, 30127393. PMC6487502 30127393

[38] Anft M. , Netter P. , Urlaub D. , Prager I. , Schaffner S. , and Watzl C. , NK Cell Detachment From Target Cells Is Regulated by Successful Cytotoxicity and Influences Cytokine Production, Cellular & Molecular Immunology. (2020) 17, no. 4, 347–355, 10.1038/s41423-019-0277-2, 2-s2.0-85072030210, 31471588. 31471588 PMC7109075

[39] Ibáñez-Molero S. , van Vliet A. , Pozniak J. , Hummelink K. , Terry A. M. , Monkhorst K. , Sanders J. , Hofland I. , Landeloos E. , Van Herck Y. , Bechter O. , Kuilman T. , Zhong W. , Marine J. C. , Wessels L. , and Peeper D. S. , *SERPINB9* is Commonly Amplified and High Expression in Cancer Cells Correlates With Poor Immune Checkpoint Blockade Response, Oncoimmunology. (2022) 11, no. 1, 2139074, 10.1080/2162402X.2022.2139074, 36465485. 36465485 PMC9710519

[40] Jiang L. , Wang Y.-J. , Zhao J. , Uehara M. , Hou Q. , Kasinath V. , Ichimura T. , Banouni N. , Dai L. , Li X. , Greiner D. L. , Shultz L. D. , Zhang X. , Sun Z.-Y. J. , Curtin I. , Vangos N. E. , Yeoh Z. C. , Geffken E. A. , Seo H.-S. , Liu Z.-X. , Heffron G. J. , Shah K. , Dhe-Paganon S. , and Abdi R. , Direct Tumor Killing and Immunotherapy Through Anti-SerpinB9 Therapy, Cell. (2020) 183, no. 5, 1219–1233.e18, 10.1016/j.cell.2020.10.045. 33242418 PMC7927154

[41] Soriano C. , Mukaro V. , Hodge G. , Ahern J. , Holmes M. , Jersmann H. , Moffat D. , Meredith D. , Jurisevic C. , Reynolds P. N. , and Hodge S. , Increased Proteinase Inhibitor-9 (PI-9) and Reduced Granzyme B in Lung Cancer: Mechanism for Immune Evasion?, Lung Cancer. (2012) 77, no. 1, 38–45, 10.1016/j.lungcan.2012.01.017, 2-s2.0-84861722676. 22387007

